# Biological Activities of *Ceratonia siliqua* Pod and Seed Extracts: A Comparative Analysis of Two Cretan Cultivars

**DOI:** 10.3390/ijms241512104

**Published:** 2023-07-28

**Authors:** Dafni-Alexandra Kavvoura, Michalis K. Stefanakis, Dimitris Kletsas, Haralambos E. Katerinopoulos, Harris Pratsinis

**Affiliations:** 1 Laboratory of Cell Proliferation and Ageing, Institute of Biosciences and Applications, NCSR “Demokritos”, 15341 Athens, Greece; dafnikavvoura@gmail.com (D.-A.K.); dkletsas@bio.demokritos.gr (D.K.); 2 Laboratory of Organic Chemistry, Department of Chemistry, University of Crete, 70013 Heraklion, Greece; michstefanakis@yahoo.gr (M.K.S.); katerinc@uoc.gr (H.E.K.)

**Keywords:** *Ceratonia siliqua*, volatile components, fatty acids, carbohydrates, antioxidant, photoprotection, tyrosinase, collagenase, advanced glycation end products, skin fibroblasts

## Abstract

*Ceratonia siliqua* L., commonly known as the carob tree, appears in most Mediterranean countries, often cultivated for the collection of its fruits to be used as food for humans and animals. This study was aimed at the phytochemical characterization of two common Cretan *C. siliqua* cultivars and the biological evaluation of deseeded pod and seed extracts regarding their putative use in cosmetics. Gas and liquid chromatographic techniques were used to assess their essential oil, fatty acid, and carbohydrate profiles. Cell-free assays, including free-radical scavenging; the inhibition of tyrosinase and collagenase; the blocking of advanced glycation end product (AGE) formation; along with assays in human skin fibroblast cultures, i.e., reactive oxygen species suppression, glutathione stimulation, and protection from oxidative stress and from ultraviolet (UVB) radiation, were also used. Extracts from both cultivars were found to possess antioxidant capacity, tyrosinase- and collagenase-inhibitory activities, an ability to block glucose-induced AGEs, and in certain cases, UVB absorbance and photoprotective activities. Seed extracts were in general more active, while the use of 30% aqueous methanol seemed to be more efficient than n-hexane for extraction. Serial partition of the most active extracts resulted in fractions with enriched biological activities. These properties make Cretan carob extracts and their fractions suitable candidates for use in cosmetics.

## 1. Introduction

*Ceratonia siliqua* L. is an evergreen tree belonging to the *Leguminosae* (*Fabaceae*) family (subfamily *Caesalpinioideae*) and it is widely known as the carob tree. Originating in the Middle East, it is widely encountered in the Mediterranean area, but it can grow in warm- or temperate-climate areas all over the world, even in arid and semiarid regions [1]. The carob tree has been domesticated and cultivated since ancient times; hence, a great variety of local cultivars exist in every country [2]. For instance, only in Southern Italy, fifty-four *C. siliqua* cultivars have been described [3]. In our days, the countries with the largest production of carob are Portugal, Italy, Spain, Morocco, Turkey, Greece, and Cyprus [4], with approximately 80% of the Greek production concentrated in the island of Crete [2].

*C. siliqua* cultivation throughout the years has aimed mainly at the collection of the fruits (carobs), but also at ornamental and other uses; e.g., nowadays, it is used against deforestation [5,6]. Carobs have the form of elongated pods (also known as locust beans) containing the pulp and the seeds (the latter usually account for 8–12% of the pod weight), while the pulp consists of a rough outer layer, the pericarp, and a soft inner layer, the mesocarp. The pulp comprises mainly sugars (mostly sucrose, and to a lesser extent glucose and fructose) and insoluble fiber (such as cellulose and hemicellulose), tannins, and other phenolic compounds (gallic acid and gallotannins), being poor in fat and protein [1,7]. The seeds contain much more protein and fewer sugars, as well as a low concentration of fat and many phenolic compounds [1,8,9].

Since ancient times, carob has been used as food for humans and animals, and during hard times like World War II in Crete or the Spanish Civil War, it has saved many lives [4,10]. Beyond its nutritional uses, carob has always had multiple pharmaceutical applications, as indicated through traditional medicinal and ethnomedicinal reports [11]. It has been proposed for glycemic control and antidiabetic activities, for combating hyperlipidemia, for anti-inflammatory effects especially in the digestive tract, for cytotoxic and anticancer effects, as well as for anxiolytic and antidepressant activities [7,12,13,14]. A less studied aspect regards the use of carob in the cosmetic industry, although the polysaccharide galactomannan (locust bean gum or E410) from the seed endosperm is already being used in the cosmetic sector, mainly due to its physical properties [15]. A recent study suggested that carob extracts, beyond their well-known antioxidant properties, also possess photoprotective activity [16].

The present study is part of an effort to support carob tree cultivation in Crete given its ecological advantages over other species, such as its very high fixation of carbon dioxide and its low requirements for water irrigation [17]. As mentioned above, many different *C. siliqua* cultivars exist in Crete, some of them imported from other countries. Based on the practical knowledge of the local producers, a typical cultivar existing in Crete for many years was selected as a primary target of the study, the so-called Imera (or Hemere) cultivar [2]. This cultivar was recently subjected to the sequencing, assembly, and annotation of its genome, which resulted in the first, nearly chromosome-level assembly for *C. siliqua* [18]. The material used for this study was collected from trees in Pines in the area of Elounda Lasithiou. Another cultivar imported from Cyprus but acclimatized in Crete, the so-called Tylliria, was also studied for reasons of comparison [2]. The material used in this study was harvested from trees in Melidochori, near Heraklion. The local producers use Imera carobs mainly for flour production and for livestock food, while Tylliria is ideal for the production of carob syrup. Extracts from the two cultivars designated CSE (*C. siliqua* Elounda) and CSH (*C. siliqua* Heraklion) were subjected to a comparative study of their activities (including antioxidant, photoprotective, cytotoxic, and enzyme-modifying ones) on skin cell cultures and in cell-free assays, towards their putative use in cosmetic and other applications. Seed extracts were studied separately from deseeded pod extracts, since the former are considered by-products of flour production that may prove useful as additives in food, pharmaceutical, and cosmetic products [19].

## 2. Results

### 2.1. Extraction and Phytochemical Analysis

#### 2.1.1. Extraction

Aqueous methanolic deseeded carob pod extracts (CSE1 and CSH1), aqueous methanolic seed extracts (CSE2 and CSH2), and n-hexane seed extracts (CSE3 and CSH3), as illustrated in Table 1, were prepared from two distinct Cretan cultivars.

#### 2.1.2. The Identification and Quantification of Essential Oils

The different components (*n* = 27) of the essential oils of the two studied samples of *C. siliqua* from Crete are listed in Table 2 according to their increasing retention times, comprising > 98% of the total. The more complex essential oil was the one obtained from CSH, with 21 compounds, followed by the sample from CSE (*n* = 17). The extracted oil was yellowish in color and sticky with a characteristic aromatic odor. The yields of the volatile fractions obtained from the pods of the two *C. siliqua* cultivars were 0.10% (CSE) and 0.11% (CSH) (*v/w*), respectively, on a dry weight basis (Table 2). Only 11 out of 27 compounds (41%) were common in the two population samples, but in different proportions. In both cases, the most predominant compounds were octanoic acid and hexanoic acid.

#### 2.1.3. Fatty Acid Composition

Fatty acids are primary metabolites that exist in every plant. Free fatty acids play a key role in human nutrition. The composition of the two studied *C. siliqua* samples from Crete is listed in Table 3.

#### 2.1.4. Carbohydrate Composition

Carob fruit bears rich nutritional value due to the presence of sugars, especially sucrose, glucose, and fructose. Table 4 reveals the sugar content (% on the basis of dry material).

#### 2.1.5. NMR Analysis of C. siliqua Extracts

**Figure 1:**
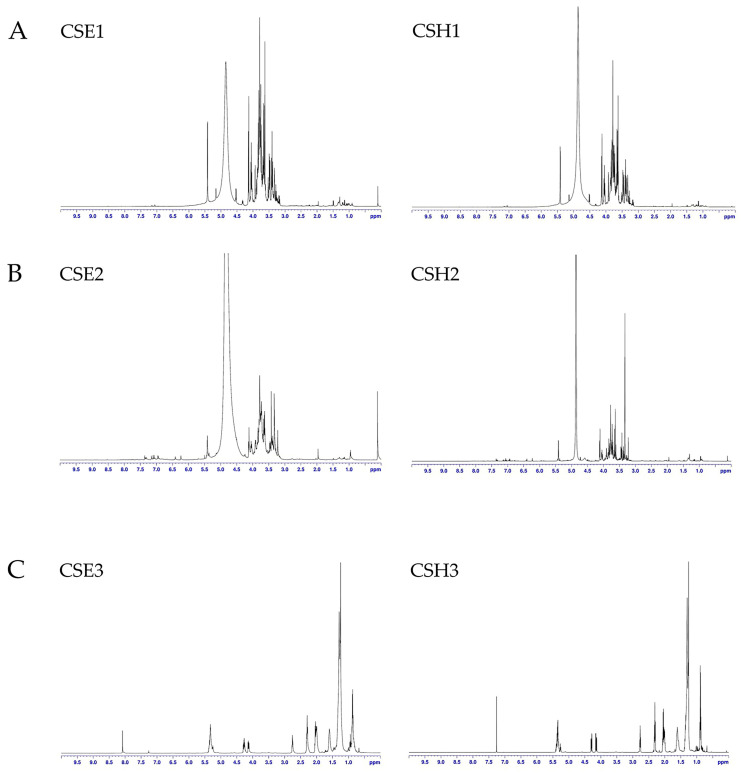
^1^H-NMR spectra from the analysis of the seed and pod extracts of *C. siliqua*: (**A**) CSE1 and CSH1 (500 MHz, CD_3_OD); (**B**) CSE2 and CSH2 (500 MHz, CD_3_OD); (**C**) CSE3 and CSH_3_ (500 MHz, CDCl_3_).

An NMR analysis of the extracts, as presented in Figure 1, revealed the different content in each extract. It is obvious that the polar extracts were rich in carbohydrates due to the signals in the region between 3.20 and 4.4 ppm (CSH1, CSE1, CSH2, and CSE2). Moreover, the polar extracts from the seeds (CSH2 and CSE2) seemed to be richer in phenolic compounds because the signals in the aromatic part of the spectrum (6.00–7.50 ppm) were fewer in the NMR from the fruits compared to those of the seeds. On the other hand, the ^1^H-NMR spectra of the hexane extracts from the seeds (CSH3 and CSE3) were rich in nonpolar compounds. The content of these extracts was mainly free fatty acids because of the signals in the aliphatic parts of the spectrum.

### 2.2. Assessment of Cell Viability following Treatment with C. siliqua Extracts

The extracts were initially evaluated for their cytotoxicity against a normal skin fibroblast cell line (AG01523) so that we could identify the noncytotoxic concentrations to be used in all subsequent cell-based assays. Two malignant cell lines, i.e., A431 epidermoid carcinoma and HT-1080 fibrosarcoma cells, were also studied in comparison with the normal skin cells. The cytotoxicity was determined 72 h after treatment with the extracts (0.32–200 μg/mL) using 3-(4,5-Dimethylthiazol-2-yl)-2,5-diphenyltetrazolium bromide (MTT), as described in the Methods section. As seen in Table S1, none of the extracts negatively impaired the viability of normal fibroblasts at concentrations ≤ 200 μg/mL. Similarly, no decreased viability of HT-1080 and A431 cells was observed following treatment with the extracts, apart from CSE3, which managed to mildly inhibit the viability of A431 cells at the highest tested dose of 200 μg/mL.

### 2.3. Antioxidant Activity of C. siliqua Extracts

**Figure 2:**
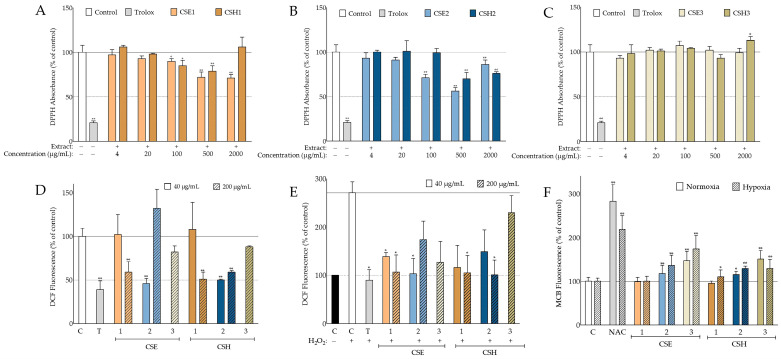
Antioxidant activity of *C. siliqua* extracts. (**A**–**C**) Comparative assessment of the radical-scavenging activity of akin extracts, from both varieties, based on the inhibition of 2,2-Diphenyl-1-Picrylhydrazyl (DPPH) absorbance 35 min postaddition (*n* ≥ 2); 6-hydroxy-2,5,7,8-tetramethylchroman-2-carboxylic acid (Trolox) (200 μM) served as the positive radical scavenger. (**D**,**E**) Intracellular antioxidant activity of carob extracts, based on 2′,7′-dichlorofluorescein (DCF) fluorescence quenching; in (**D**), basal intracellular reactive oxygen species (ROS) levels of normal human fibroblasts (AG01523) 210 min after 2′,7′-dichlorodihydrofluorescein diacetate (DCFH-DA) addition are depicted (*n* = 3); in (**E**), elevated ROS levels an hour after H_2_O_2_ addition, at a final concentration of 160 μΜ, are shown (*n* = 2); in both (**D**,**E**), vehicle (DMSO) served as negative control (C) and Trolox (T) at 100 μM served as positive control. (**F**) Intracellular glutathione (GSH) levels of human fibroblasts pretreated with carob extracts at the highest noncytotoxic dose of 200 μg/mL (Table S1) under normal oxygen tension and hypoxic conditions, respectively (*n* ≥ 3); vehicle (DMSO) served as negative control (C) and N-acetylcysteine (NAC) at 10 mM served as positive control. Bars represent mean values ± SD. * *p* < 0.05; ** *p* < 0.01 (Student’s *t*-test). Control values were set to 100%.

The extracts’ antioxidant activity was then studied in a cell-free system, based on their ability to scavenge the free radical 2,2-Diphenyl-1-Picrylhydrazyl (DPPH). Most of the extracts tested showed some degree of radical-scavenging capacity (as shown in Figure 2A–C). Deseeded carob fruit and seed aqueous methanolic extracts, from both cultivars, exhibited statistically significant antioxidant activity in a variety of different concentrations ranging from 2000 μg/mL to 100 μg/mL. On the other hand, n-hexane seed extracts were found to be the least active, presenting marginal, not statistically significant activity. The most promising were the carob seed aqueous methanolic extracts, with CSE2, at 500 μg/mL, decreasing the DPPH radical absorbance to 56% of the negative control. Interestingly, CSE2 showed higher antioxidant potential at 500 μg/mL in comparison to the higher concentration of 2000 μg/mL, suggesting the coexistence of antioxidant and pro-oxidant molecules. Furthermore, this pro-oxidant activity may be developed in a time-dependent manner, since higher concentrations were found at first to strongly lower the DPPH absorbance compared to the control, but progressively, their efficiency declined.

Our findings regarding the DPPH-radical-scavenging capacity of the carob extracts were promising. On the other hand, this is a cell-free assay and provides no insight on the complex mechanisms that take place intracellularly. As an example, cell membrane permeability can hinder the intracellular activity of a compound with high antioxidant potential [20]. Thus, we evaluated the carob extracts’ abilities to quench 2′,7′-dichlorofluorescein (DCF) fluorescence. 2′,7′-dichlorodihydrofluorescein diacetate (DCFH-DA) is a nonfluorescent compound that upon endocytosis, is subjected to hydrolysis yielding nonfluorescent dichlorodihydrofluorescein (DCFH). Various oxidants can interact with DCFH and oxidize it to fluorescent DCF [21]. For this purpose, normal human fibroblasts (AG01523), pretreated with two distinct noncytotoxic doses of the extracts (200 and 40 μg/mL, selected based on the MTT assay; Table S1), were evaluated for their DCF fluorescence both in normal ROS conditions and upon stimulation with H_2_O_2_. CSE3 and CSH3 were tested only at the highest concentration, given their lack of any antioxidant activity at any concentration tested in the DPPH assay (Figure 2C). The best intracellular antioxidant activity on the basal ROS levels was obtained 210 min after the addition of DCFH-DA and can be seen in Figure 2D. For the stimulated ROS levels, the results obtained an hour after adding H_2_O_2_ are presented in Figure 2E, since at this time point, a sufficient increase in oxidative stress (2.7-fold) and high antioxidant activity of the tested extracts were observed. In both the basal levels and in the stimulated ones, antioxidant capacity seemed to increase with concentration. The only exception was CSE2, which was found to be more efficient at lower concentrations, as previously observed in the DPPH assay. Indeed, CSE2 at 40 μg/mL exhibited an antioxidant potential similar to the positive control. Carob pod aqueous methanolic extracts, CSE1 and CSH1, at 200 μg/mL were also found to strongly inhibit DCF fluorescence in both basal and stimulated conditions, comparably to Trolox. The hexane seed extracts were the least active, exhibiting only marginal antioxidant activities. Overall, these findings are in accordance with the DPPH assay described previously.

Glutathione (GSH) is a multivalent antioxidant of the cell. Some of its actions include the direct scavenging of radicals, acting as a cofactor to antioxidant enzymes and replenishing oxidized antioxidants [22]. Therefore, we sought to investigate the effect of the highest noncytotoxic doses of carob extracts (Table S1) on the GSH levels of AG01523 fibroblasts by measuring the fluorescence of monochlorobimane (MCB). MCB is a highly selective fluorescent compound when conjugated with the thiol group of GSH and can be used to detect GSH intracellularly upon internalization [23]. Our findings can be seen in Figure 2F. Most of the cells treated with carob extracts exhibited elevated GSH levels regardless of oxygen tension. The best activity was observed with CSE3 that managed to elevate the fluorescence of MCB to 147 and 174% in normal and hypoxic conditions, respectively. The corresponding extract from the Heraklion cultivar was found to be comparably active. CSE2 and CSH2 were the second most impactful, managing to increase intracellular glutathione significantly in both conditions. The fruit extracts did not produce any effect apart from CSH1 under low oxygen tension.

### 2.4. Western Blot Analysis of Signaling Molecules

Due to the promising antioxidant activities exhibited by the extracts under oxidative stress, we performed a Western blot analysis to assess their effect on protein kinase B (Akt) and AMP-activated protein kinase (AMPK) phosphorylation under H_2_O_2_-induced oxidative stress. Akt modulates many functions including metabolism, cell growth, proliferation, and survival [24]. AMPK, on the other hand, regulates cell growth and metabolism under low-ATP conditions [25].

**Figure 3:**
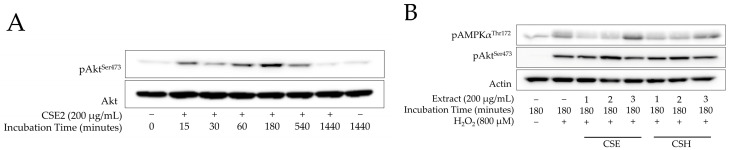
Western blot analysis. (**A**) Normal fibroblasts were preincubated with 200 μg/mL of CSE2 for various incubation times and total protein was extracted; Western blot analysis was performed for phospho-Akt^Ser473^ and the nonphosphorylated form (Akt) to verify equal loading. (**B**) Cells preincubated with 200 μg/mL of carob extracts for 180 min were treated with H_2_O_2_ (800 μΜ) for 45 min before collection and Western blot analysis for phospho-Akt^Ser473^ and phospho-AMPKα^Thr172^, respectively; Actin was used to verify equal loading. Representative images of two independent experiments are presented.

Since CSE2 showed the most promising antioxidant potential, we incubated AG01523 cells with the highest noncytotoxic concentration (Table S1) and collected cell lysates at various time points. By performing a Western blot analysis for p-Akt, we found a time-dependent increase in Akt phosphorylation that appeared after 15 min of incubation in the presence of the extract and lasted for at least 9 h (Figure 3A). The strongest signal was provided by the cell lysates collected 180 min after the addition of the extract. Using that as a guide, we evaluated the effects of all carob extracts on AMPK and Akt phosphorylation under H_2_O_2_-mediated oxidative stress. It can be seen in Figure 3B that treatment with H_2_O_2_ stimulated p-AMPKα in comparison to the unchallenged control. Furthermore, in cells incubated with CSE3, increased AMPKα phosphorylation was observed in comparison to the H_2_O_2_-treated cells used as a control, whilst all other extracts led to a decrease in p-AMPKα. Oxidative stress was found to produce a stimulating effect on Akt phosphorylation as well, with all extracts, except CSE1 and CSE3, further reinforcing the signal. A densitometric analysis of the blots is available in Figure S1.

### 2.5. Photoprotective Activity of C. siliqua Extracts

**Figure 4:**
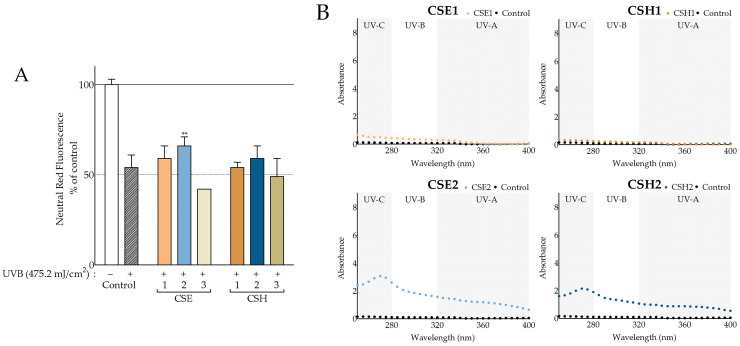
Photoprotective activity of *C. siliqua* extracts. (**A**) Photoprotective activity of carob extracts on ultraviolet B (UVB)-irradiated normal human fibroblasts; the highest noncytotoxic concentration was tested (200 μg/mL, Table S1); DMSO-treated, irradiated, and nonirradiated cells served as negative controls; bars represent mean values ± SD (*n* ≥ 3); error bars correspond to statistical difference between irradiated control and irradiated samples; ** *p* < 0.01 (Student’s *t*-test); control values were set to 100%. (**B**) Carob extracts’ UV absorbance scans at 200 μg/mL in comparison to phosphate-buffered saline (PBS).

Ultraviolet B (UVB) irradiation can compromise skin integrity by increasing intracellular oxidative stress upon the photosensitization of chromophores, causing DNA damage, subduing immune responses, and promoting apoptosis and carcinogenesis [26,27]. For this purpose, we assessed the viability of irradiated normal fibroblasts that had been treated with the extracts before, during, and after irradiation. The UVB dose applied was 475 mJ/cm^2^, enough to produce an approximate 50% reduction in viability in the UVB-treated control cells [28]. Additionally, we measured the extracts’ UV absorption spectra and determined their sun protection factor (SPF) as described by Mansur [29]. As presented in Figure 4A, CSE2 was found to significantly increase the viability of irradiated fibroblasts 72 h after the UVB treatment. Furthermore, it appeared to possess UV-absorbing capacity (Figure 4B) and an SPF value of 18.19 (Table 5). CSH2, despite having an SPF of 13.07 and displaying mild absorbance in the UVB region, was not able to alter the survival of the cells significantly. CSE1 and CSH1 appeared to have a marginal, not statistically significant, positive effect on the irradiated cells but had no irradiation-absorbing properties. It is important to note that even though UV-absorbing abilities can play a crucial role by shielding cells, other factors may contribute to photoprotective effects, since the cells were not only irradiated in the presence of the extracts but also treated with them before and after exposure to irradiation.

### 2.6. Inhibitory Activity of C. siliqua Extracts against Tyrosinase, Collagenase, and Advanced Glycation End Products

Tyrosinase plays an important role in melanogenesis [30], whilst collagenase [31] and advanced glycation end products (AGEs) [32] play important roles in skin ageing. Therefore, we assessed the potential enzyme-altering and AGE-formation-inhibitory activities of the extracts in cell-free assays.

**Figure 5:**
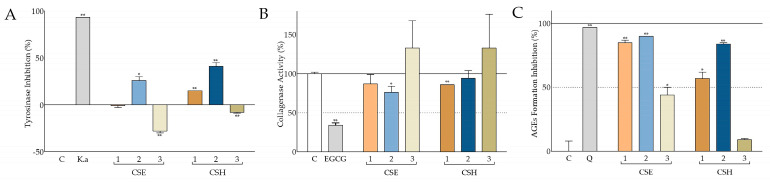
Inhibitory activity of *C. siliqua* extracts against tyrosinase, collagenase, and advanced glycation end products. (**A**) Tyrosinase inhibitory activity of the carob extracts at 200 μg/mL; kojic acid (K.a) at 0.1 mM was used as the reference compound. (**B**) Percentage of collagenase activity in the presence of carob extracts at 200 μg/mL; (−)-epigallocatechin gallate (EGCG) at 1 mM served as the positive control. (**C**) Advanced glycation end product (AGE) formation inhibition by the carob extracts at 200 μg/mL; quercetin (Q) at 662 μΜ served as the positive inhibitor. Vehicle (DMSO) served as negative control (C) in all assays. Control values were set to 0% or 100%. Bars depict mean values ± SD (*n* = 2). * *p* < 0.05; ** *p* < 0.01 (Student’s *t*-test).

Their effect on tyrosinase was evaluated using mushroom tyrosinase and L-3,4-dihydroxyphenylalanine (L-DOPA) as a substrate, and the results are presented in Figure 5A. CSE2 and CSH1-2 exhibited various degrees of tyrosinase inhibition, whilst CSE3 and CSH3 were found to induce the opposite effect. CSH2 produced the highest inhibition, reaching 41 ± 4%. CSE2 and CSH1 were also found to inhibit tyrosinase to a lesser, yet significant, extent. 

The effect of the extracts on collagenase activity was assessed using *Clostridium histolyticum-*derived collagenase and a selective substrate that fluoresces upon enzymatic cleavage. The results are presented in Figure 5B. CSE3 and CSH3 had no inhibitory result in collagenase activity. CSE1-2 and CSH1-2 exhibited anticollagenase activity, which was statistically significant for CSE2 and CSH1, inhibiting the enzyme activity to 76 ± 8 and 86 ± 0%, respectively.

Bovine serum albumin (BSA) fraction V was used to monitor the inhibitory effect of the carob extracts on glucose-induced advanced glycation end products (AGEs). Our findings, as illustrated in Figure 5C, show that all carob extracts, except from CSH3, significantly inhibited AGE formation. CSE1-2 and CSH1-2 were able to prevent more than 50% of AGE formation, with CSE1 and CSE2 producing an intense effect: 85 ± 2 and 90 ± 0%, respectively, comparable to the reference compound, quercetin.

### 2.7. Fractionation of C. siliqua Pod and Seed Extract

As mentioned above (Introduction), the primary target of the study was the Imera cultivar. Given the promising antioxidant activities of CSE1 (deseeded pod) and CSE2 (seeds) extracts, as well as the photoprotective and enzyme-modifying activity of CSE2, they were subjected to serial partition with four different solvents of increasing polarity, namely n-hexane, ethyl acetate (EtOAc), and butanol (BuOH), keeping the aqueous residue of the last elution (Table 6).

### 2.8. Assessment of Cell Viability following Treatment with C. siliqua Pod and Seed Extract Fractions

The obtained fractions were also screened for their cytotoxicity on the previously mentioned cell lines in a variety of different concentrations ranging from 200 μg/mL to 0.32 μg/mL (Table S2). CSE2.1 exhibited statistically significant cytotoxic effects on normal fibroblasts at 200 μg/mL. Consequently, the next highest concentration, 40 μg/mL, assessed as not affecting viability, was chosen for the succeeding experiments. Additionally, CSE1.2 was found to negatively affect normal fibroblasts; thus, the highest noncytotoxic dose was significantly lower, at 10 μg/mL. On the other hand, CSE2.2, whilst noncytotoxic to AG01523 fibroblasts under normal serum conditions, was found to affect viability in a low-serum or a serum-free environment. This dependance on serum conditions was not reflected in the MTT assay since it was performed with 15% (*v*/*v*) serum, but was observed upon microscope inspection in serum-free protocols. Based on that, the highest noncytotoxic concentration was either 200 or 40 μg/mL depending on the protocols’ serum demands. Fractions were also tested against cancer cells, similarly to the extracts. The hexane fractions, CSE1.1 and CSE2.1, effectively obliterated both epidermoid carcinoma (95% and 94%, respectively) and fibrosarcoma cells (98% and 99%, respectively) at a concentration of 200 μg/mL. The half-maximal inhibitory concentration (IC_50_) values for both cancer cell strains were below 100 μg/mL. CSE1.2 and CSE2.2, the fractions obtained with ethyl acetate, also inhibited cancer cell growth, with CSE1.2 displaying the lowest IC_50_ values of 28 ± 6 μg/mL against A431 and 31 ± 9 μg/mL against HT-1080. Butanol and aqueous fractions were not found to be cytotoxic to either normal or cancer cells, apart from CSE1.3 against fibrosarcoma cells (IC_50_: 135 ± 36 μg/mL).

### 2.9. Antioxidant Activities of C. siliqua Pod and Seed Extract Fractions

**Figure 6:**
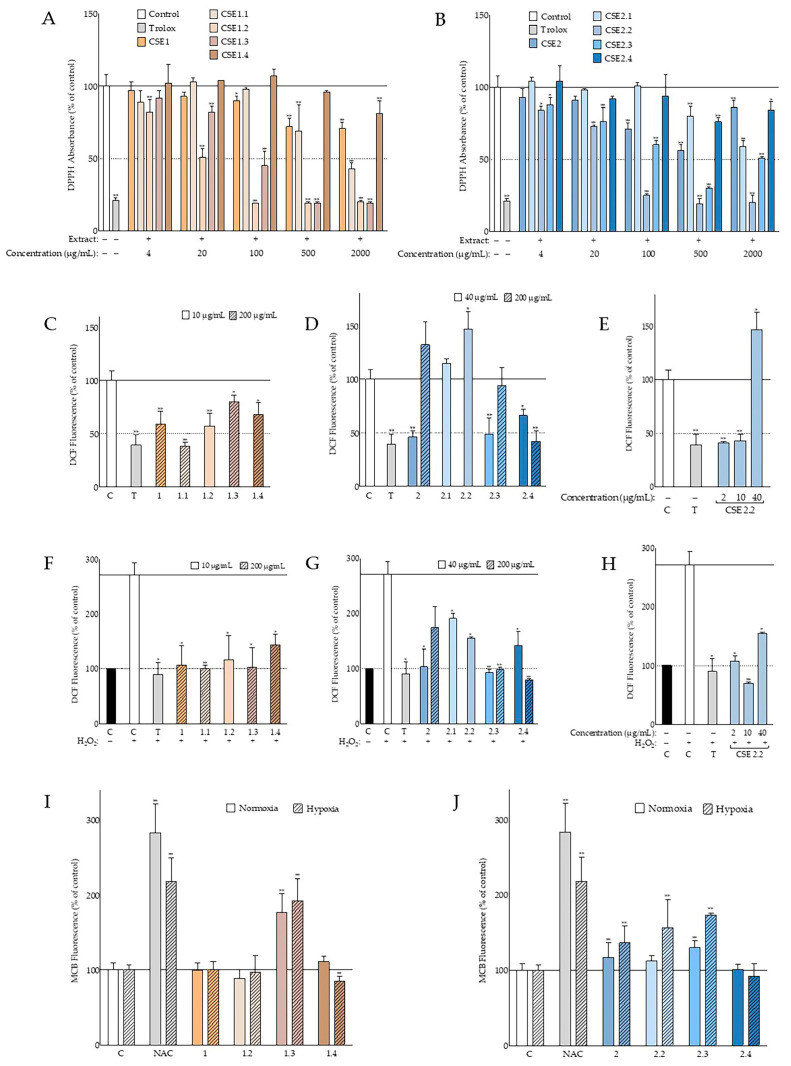
Antioxidant activity of CSE fractions. (**A**,**B**) DPPH-radical-scavenging activity of the fractions in comparison to the extract at 35 min after DPPH solution addition (*n* ≥ 2); vehicle (DMSO) and Trolox (200 μM) served as negative and positive controls, respectively. Intracellular (AG01523) antioxidant activity of carob fractions towards basal ROS levels (**C**–**E**) 210 min after DCFH-DA addition (*n* = 3) and towards elevated ROS levels (**F**–**H**) an hour after H_2_O_2_ (160 μM) addition (*n* = 2); DMSO-treated cells served as controls (C) and Trolox (T) at 100 μΜ served as the reference compound. (**I**,**J**) Intracellular GSH levels of human fibroblasts pretreated with carob extracts and their fractions, at the highest noncytotoxic concentration (Tables S1 and S2), under normal oxygen tension and hypoxic conditions, respectively (*n* ≥ 3); DMSO-treated cells served as controls (C) and NAC (10 mM) served as positive control. Bars represent mean values ± SD. * *p* < 0.05; ** *p* < 0.01 (Student’s *t*-test). Control values were set to 100%.

The methods previously applied for the extracts were also used with the fractions. Regarding antioxidant activities, the results are presented in Figure 6. As can be seen in Figure 6A,B, when comparing the antioxidant activities of the fractions to the extracts, the scavenging potential improved with fractionation. In both cases, the aqueous residues were the least active followed by the fractions partitioned with n-hexane. Having said that, most of them were able to reduce the absorbance of the DPPH radical significantly in the two highest concentrations tested, with CSE1.1 scavenging more than 50% of the radical (IC_50_: 1177 ± 240 μg/mL). Ethyl acetate and butanol fractions, on the other hand, exhibited high antioxidant potential, even in lower concentrations. CSE1.2 and CSE2.2 had the best activity in all the concentrations tested and the lowest IC_50_ values of 17 ± 1 and 35 ± 5 μg/mL, respectively. Their activity was comparable to that of Trolox for most of the concentrations tested. Butanol-partitioned fractions, CSE1.3 and CSE2.3, were also found to be potent antioxidants since IC_50_ values of 79 ± 22 and 140 ± 68 μg/mL were recorded, respectively. Interestingly, similarly to the extract, CSE2.3 and CSE2.4 time-dependently exhibited superior DPPH-radical-scavenging capacity at 500 μg/mL to that at 2000 μg/mL. Thus, it is possible that the pro-oxidant effect of the aqueous methanolic carob fruit extract can be attributed to compounds present in the butanol fraction and the aqueous residue.

When examining the effect of CSE1 fractions, at the highest noncytotoxic concentrations (Table S2), on intracellular ROS levels, we found that like the extract, CSE1.1 and CSE1.2’s reducing capacities were comparable to those obtained with the reference compound, Trolox (Figure 6C). Furthermore, CSE1.1 managed to reduce DCF fluorescence more effectively than the extract. Overall, all fractions managed to reduce ROS levels significantly. Lower concentrations of CSE1.2 and CSE1.3, as presented in Figure S2, were equally active, while CSE1, CSE1.1, and CSE1.4 were significantly less effective. After exogenous ROS production stimulation (Figure 6F), all fractions presented significant antioxidant activity, comparable to that of the extract and the positive control, CSE2.

CSE2.2 and CSE2.3, shown in Figure 6D,E, exhibited greater potential when applied at lower doses. CSE2.2 strongly inhibited ROS formation at the doses of 2 and 10 μg/mL (Figure 6E). CSE2.3 and CSE2.4, at the most effective dose, appeared to strongly quench fluorescence to a similar degree to Trolox. In H_2_O_2_-challenged cells, although most extracts were beneficial, CSE2.3 and CSE2.4 were the most effective along with CSE2.2 at lower doses (Figure 6G,H). Interestingly, they managed, at their optimal concentration, to reverse the ROS stimulation produced by H_2_O_2_ and suppress oxidative stress levels below unstimulated levels. However, the added reduction was not statistically significant. 

Even though CSE1 did not impact GSH levels, as seen in Figure 6I, CSE1.3 effectively stimulated GSH levels under both oxygen tension conditions. Moreover, in hypoxia, the increase in GSH was comparable to the increase obtained from the positive control, N-acetylcysteine. It is possible that the effectiveness of CSE1.3 is due to enrichment in the compound(s) responsible, compared to their content in the extract, that is below the threshold necessary to induce GSH. Regarding fractions partitioned from CSE2 (Figure 6J), most of them elevated MCB fluorescence more than the extract. CSE2.2, and especially CSE2.3, were the most active. Hexane fractions from both extracts affected cell adherence under the experiment conditions applied and could not be included.

### 2.10. Photoprotective Properties of C. siliqua Pod and Seed Extract Fractions

**Figure 7:**
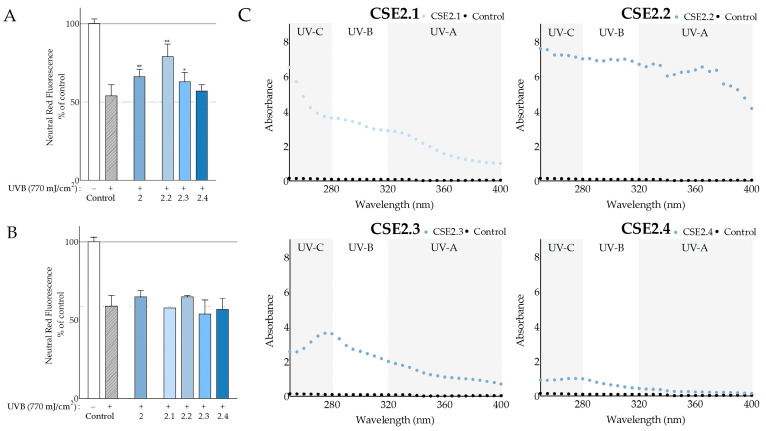
Photoprotective properties of *C. siliqua* pod and seed extract fractions. Photoprotective activity of CSE2 and its fractions on UVB-irradiated normal human fibroblasts at 200 μg/mL (**A**) and 40 μg/mL (**B**); DMSO-treated, irradiated, and nonirradiated cells served as controls; bars represent mean values ± SD (*n* ≥ 3); error bars correspond to statistical difference between irradiated control and irradiated samples; * *p* < 0.05; ** *p* < 0.01 (Student’s *t*-test); control values were set to 100%. (**C**) UV absorbance scans of CSE2 fractions at 200 μg/mL in comparison to PBS.

Due to the photoprotective effects displayed by CSE2, we sought to assess the possible beneficial effects of its fractions. When evaluating their effect at the highest noncytotoxic doses (Table S2) of 200 μg/mL, we found that CSE2.2 and CSE2.3 had the most ameliorating effect on irradiated fibroblasts, increasing the viability of the UVB-treated control from 54 ± 7 to 79 ± 8 and 63 ± 6%, respectively (Figure 7A). Moreover, the CSE2.2-treated cells had significantly higher viability compared to the CSE2-treated cells. CSE2.2 also exhibited high UV-absorbing abilities (Figure 7C) and an SPF value of 69.87 (Table 7). CSE2.3 also absorbed in the UV region and its SPF was calculated at 25.06. On the other hand, CSE2.4 did not provoke a considerable outcome on any of the parameters evaluated. CSE2.1 was cytotoxic at 200 μg/mL and was tested along with the other fractions at 40 μg/mL under the same conditions. However, none were significantly effective at this dose, as can be seen in Figure 7B.

### 2.11. Inhibitory Activity of CSE Fractions against Tyrosinase, Collagenase, and Advanced Glycation End Products

**Figure 8:**
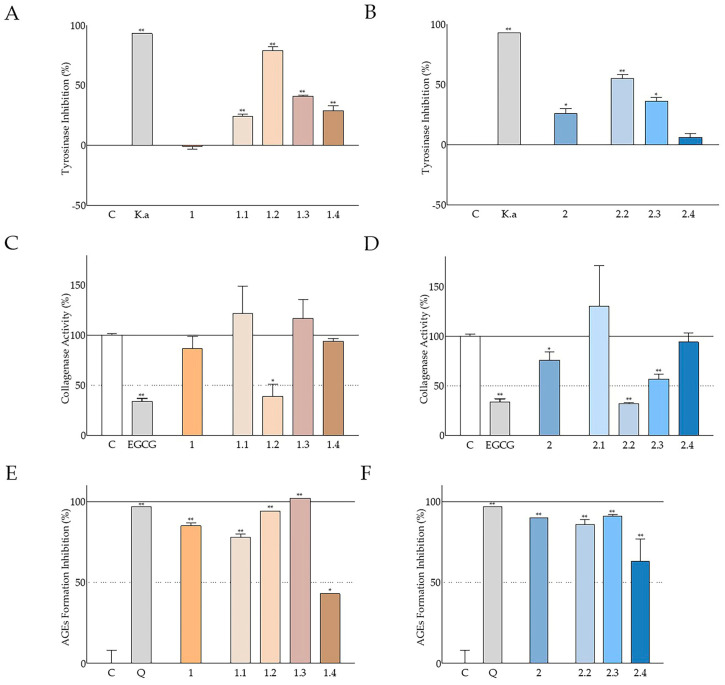
Antityrosinase, anticollagenase, and AGE-inhibitory activity of the CSE fractions. (**A**,**B**) Tyrosinase-inhibitory activity of the carob extracts and their fractions at 200 μg/mL; vehicle (DMSO) served as control (C) and kojic acid (K.a) at 0.1 mM served as the reference compound; control values were set to 0%. (**C**,**D**) Effect of carob extracts and fractions on collagenase activity at a final concentration of 200 μg/mL; DMSO served as control (C) and EGCG (1 mM) served as the reference compound; control values were set to 100%. (**E**,**F**) Inhibitory activity of extracts in comparison to their fractions on AGE formation (200 μg/mL); DMSO served as control (C) and quercetin (Q) at 662 μM served as the positive control; control values were set to 0%. Bars illustrate mean values ± SD (*n* = 2). * *p* < 0.05; ** *p* < 0.01 (Student’s *t*-test).

When examining the potential enzyme- and AGE-inhibitory activities of the fractions illustrated in Figure 8, we made the following observations. Even though carob fruit extract showed no activity in the assay, all of its fractions were found to exhibit significant antityrosinase effects, with CSE1.2 eliciting the most prominent inhibition of 79 ± 3% (Figure 8A). Similarly, greater inhibition was observed with the fractions in comparison to the aqueous methanolic carob seed extract. CSE2.2 (Figure 8B) caused a decline of 55 ± 3%, while CSE2.3 was less active (36 ± 3%). Due to the scarcity of the CSE2.1 fraction, it was omitted from this experiment. Overall, the fractions with the best antityrosinase activity were the ones partitioned with ethyl acetate and butanol for both the fruit and the seed aqueous-methanol extract.

As displayed in Figure 8C, the ethyl acetate fraction of the carob fruit extract was found to be responsible for the marginal anticollagenase activity of the extract. Furthermore, it produced a similar reduction to the reference compound of 39 ± 12%. The same observation was made for the corresponding fraction of the seed extract, CSE2.2, which lowered the collagenase activity to 32 ± 1% (Figure 8D). This effect was not only comparable but marginally greater than that of (−)-epigallocatechin gallate (EGCG). CSE2.3, a butanol fraction, was also found to strongly decrease enzyme activity.

All fractions successfully inhibited AGE formation, as seen in Figure 8E,F. Ethyl acetate and butanol fractions of the fruit extract managed to surpass the activity of the extract, with CSE1.3 annihilating all AGE formation. Fractions of the seed extract were found to have an analogous capacity to the extract. On both accounts, aqueous residues from the fractionation exhibited a significant, yet lower, inhibition comparatively to the rest.

## 3. Discussion

The present study focuses on the comparative analysis of carob extracts obtained from two distinct *C. siliqua* cultivars encountered in different geographical locations on the island of Crete. It has been shown previously that gender and cultivar significantly influence the chemical content and the biological activities of carob extracts with Portuguese origin [33]. This also seems to be the case with the two Cretan cultivars of the present study, at least concerning the chemical content; only 11 out of 27 essential oil compounds (41%) were found to be common in the pods of the two cultivars, while also the fatty acid and the carbohydrate composition varied considerably (Table 2, Table 3 and Table 4). The higher sucrose content of CSH compared to CSE reflects the knowledge of the local producers, who prefer the first cultivar for the production of carob syrup. As far as we know, there is only one study concerning the fatty acid and carbohydrate content of Cretan carob fruits—without, however, more information on the selected cultivars—assessing their composition in three different growth stages, the second one being the most appropriate for comparison with our results, based on the collection period of the plant material [34]. Our results are in line with the above study concerning palmitic acid, but there are certain differences regarding ω3 and ω6 fatty acids since we detected lower amounts of linolenic acid and higher percentages of the linoleic one (Table 3). Moreover, our results agree regarding the content of the main carob carbohydrate (sucrose), with minor differences in the glucose and fructose contents. Still, the fatty acid and carbohydrate contents determined in both studies are within the limits that have been generally described in the literature [35,36].

The extracts from the two Cretan cultivars were further subjected to a comparative study of a series of biological activities related to their potential for use in cosmetics. Their effect on human skin fibroblast viability was evaluated and none of the carob extracts exhibited cytotoxicity up to the concentration of 200 μg/mL (Table S1). On the other hand, some of the fractions, i.e., CSE1.2 and CSE2.1 (under serum-free and low-serum conditions), did result in reduced viability; hence, lower noncytotoxic concentrations were chosen for the respective experiments (Table S2). We did not locate any other studies in the literature regarding carob extracts’ effects on human skin fibroblasts; however, liposome encapsulated *C. siliqua* pod extracts tested on 3T3 embryonic mouse fibroblasts, and immortalized human keratinocytes (HaCaT) have been reported to be noncytotoxic [37], in accordance with our findings. There are many studies in the literature regarding carob extracts’ cytotoxicity towards a variety of different cancer cell lines [33,38,39,40,41,42,43,44,45,46,47,48,49,50,51]. Cytotoxic activity has been mainly found with polar extracts and is believed to be a result of synergy between many compounds contained in carob [7,44]. Although not directly related to the scope of the present study, we tested the extracts from the two Cretan cultivars on human epidermoid carcinoma (A431) and human fibrosarcoma (HT-1080) cells. CSE3 at the highest concentration tested was marginally cytotoxic for A431 cells, while fraction CSE1.2, and to a lesser extent fractions CSE1.1 and CSE2.1, were cytotoxic for both cell lines (Tables S1 and S2). A 30% aqueous methanolic leaf extract from the Imera (Elounda) cultivar was also cytotoxic for HT-1080 fibrosarcoma cells (Table S3). Regarding the different efficacies observed among the various fractions, these may be related to the amount of carbohydrates, such as sucrose, which may exert protective effects, counteracting the cytotoxic activities of other constituents [40,52]. The cytotoxic activities of Cretan carob extracts and their fractions against cancer cells are being further investigated in a separate study.

A main target of our study was the assessment of the antioxidant activity of the extracts from the two Cretan carob tree cultivars. The antioxidant activity of C. *siliqua* extracts was extensively investigated in cell-free systems. Previously, higher DPPH radical scavenging has been reported with methanolic carob leaf extracts compared to pulp ones, as well as a correlation between antioxidant activity and gender [53]. Leaf decoctions were also found to be more active than the pulp and seed ones [54], as well as more potent in total flavonoids, tannins, and phenols in comparison to the pulp [55]. Their results are in accordance with our findings since carob leaf 30% aqueous methanolic extract, CSE4, from the Imera cultivar (Elounda) was the only extract capable of reducing DPPH absorbance more than 50%, hence allowing the calculation of its IC_50_ at 294 ± 35 μg/mL (Table S3). On the other hand, the scavenging abilities of aqueous and methanolic carob leaf extracts from Turkey have been reported to be higher than the above calculated for the Imera cultivar [56]. Regarding the role of the solvent selected for extraction, our results from the seed extracts of both studied cultivars indicate that polar solvents are more efficient for the recovery of the DPPH-scavenging activities (Figure 2B vs. 2C), in agreement with previous studies using DPPH or a similar method, i.e., 2,2′-azino-bis[3-ethylbenzthiazoline-6-sulphonic acid] (ABTS) [57,58]. In these studies, as well as in the present one, inferior activity for hexane carob pulp and/or seed extracts compared to aqueous and methanolic ones was observed. Other solvent systems, such as 80% aqueous acetone [59,60], have also been reported to be very efficient for deseeded carob pod extraction.

In general, the IC_50_ values reported in the literature for methanolic carob pod extracts vary from around 100–200 μg/mL [61] to around 10 mg/mL [33], and this may be due to the different geographical origins, the cultivar, the gender, or the ripening stage [62]. Regarding the two Cretan cultivars presented here, the reduction in DPPH absorbance for CSE1 and CSH1 (Figure 2A) was not sufficient to calculate an IC_50_ (the highest concentration tested being 2 mg/mL); however, IC_50_ values of 1177 ± 240, 17 ± 1, and 79 ± 22 μg/mL were obtained from fractions CSE1.1, CSE1.2, and CSE1.3, respectively (Figure 6A). It is important to note that the carobs used in this study had reached maturity, while more potent antioxidant activities have been reported for unripe carob pulp extracts than ripe ones [49,60,63,64]. On the other hand, ethyl acetate ripe seed extracts had lower IC_50_ values when compared to the unripe ones [49]. Regarding the carob seed extracts, the best antioxidant activity was exhibited by the 30% aqueous methanolic extract CSE2 that managed to decrease DPPH radical absorbance to 56% of the negative control at 500 μg/mL and at 71% at 100 μg/mL (Figure 2B). This efficacy was comparable to other studies based on Moroccan carob seeds [65] but inferior to another using carob seeds from Algeria [66]. However, the fractions CSE2.2 and CSE2.3 of the seed extract yielded much better IC_50_ values (35 ± 5 and 140 ± 68 μg/mL, respectively; Figure 6B).

The promising results obtained from the cell-free assay led us to evaluate the antioxidant capacity of the carob extracts under study in the cellular context. Indeed, the extracts from both Cretan cultivars proved to be efficient in suppressing not only the basal ROS levels in human skin fibroblast cultures, but also the increase in ROS levels provoked by hydrogen peroxide (Figure 2D,E). Certain extracts and fractions were found to be as potent as the positive control (Trolox) (Figure 2D,E and Figure 6C–H). The inferior antioxidant activity following extraction with hexane compared to the 30% aqueous-methanol system, that was observed in the DPPH experiments, was also supported by the intracellular ROS levels. Our results agree with findings in the literature, where pretreatment with *C. siliqua pod* extract was shown to ameliorate the viability of 3T3 embryonic mouse fibroblasts, immortalized human keratinocytes, and A431 squamous carcinoma cells exposed to H_2_O_2_ [37], while an aqueous carob pod extract was found to protect against H_2_O_2_-induced DNA damage [42]. Interestingly, reducing effects of carob extract on intracellular ROS levels have been also reported for human sperm cells [67]. Extracts from other parts of the carob tree, such as sapwood and leaf, have been shown to revert H_2_O_2_-stimulated intracellular ROS levels of HeLa cells [51,51], though germ flour extract was observed to increase ROS levels in the same cell type [47]. To our knowledge, this is the first study reporting the effects of *C. siliqua* extracts and fractions on basal and H_2_O_2_-challenged ROS levels of human skin fibroblasts.

A further approach for assessing the antioxidant properties of the carob extracts was the evaluation of the intracellular GSH levels (Figure 2D and Figure 6I,J). Both seed extracts were found to increase the GSH levels of human skin fibroblasts, as well as some of the fractions tested. The fact that hexane seed extracts CSE3 and CSH3—that are expected to contain fatty acids, among others—exhibited high activities in this assay may be related to previous observations that the exposure of endothelial cells to fatty acids for over 12 h leads to an increase in GSH levels [68]. In general, under hypoxic conditions, the percentage of GSH stimulation was higher compared to normoxic ones, most probably due to the fact that hypoxia minimized basal GSH levels, as previously shown in various cell types [69,70]. Although there are several reports on the beneficial effects of various carob extracts on the GSH levels in whole organisms, mainly as part of their antioxidant defenses and during detoxification processes [71,72,73,74], this is the first study showing that *C. siliqua* extracts stimulate GSH at the cellular level.

The above protective effects of the Cretan carob extracts against ROS and oxidative stress were also evident when the activation of two signaling molecules, i.e., PKB/Akt and AMPKα was examined. Akt is considered a key molecule promoting cell survival [24], while AMPK is characterized as a guardian of metabolism and mitochondrial homeostasis [75]. AMPK is a downstream target of Akt activation; however, it has been recently shown that AMPK itself can activate Akt under cellular stress conditions [76]. We observed that the treatment of human skin fibroblasts with carob seed extract induced Akt activation (Figure 3A), suggesting an augmentation of the survival cell mechanisms. Pretreatment of the cells with seed extracts from both Cretan *C. siliqua* cultivars enhanced the subsequent H_2_O_2_-induced Akt activation (Figure 3B), further supporting cell survival, while on the other hand, it suppressed AMPKα phosphorylation. Other natural products with antioxidant and survival activities have been also shown to suppress H_2_O_2_-induced AMPKα phosphorylation [77]. Interestingly, the extracts obtained with hexane as a solvent (CSE3 and CSH3), which had shown inferior antioxidant capacity in the DPPH and DCFH-DA assays compared to the 30% aqueous methanolic ones (CSE2 and CSH2; Figure 2), were also unable to suppress AMPKα phosphorylation (Figure 3B). Interestingly, a recent study reported that in a different cellular context, i.e., in breast cancer cells, carob extracts showed the ability to suppress Akt phosphorylation, hence suppressing the detrimental cancer cell survival [49].

Beyond the antioxidant properties, the ability of the carob extracts to protect against UV radiation was evaluated, being a feature of great importance to cosmetics. This was assessed at the level of simple UV-screening, as well as at the more complicated level of conferring protection against UV-induced cytotoxicity. The screening capacity was found to be enhanced in aqueous methanolic seed extracts of both cultivars (Figure 4B) compared to deseeded pod extracts, in agreement—at least qualitatively—with observations based on plant material from other countries [78]. Overall, the aqueous methanolic seed extracts from both Cretan cultivars absorbed more than the hexane seed extracts. A recent study on the effects of processing in ethanolic *C. siliqua* pod extracts regarding their antioxidant and photoprotective potential [16] included a calculation of in vitro SPF, which ranged from 8.62 to 22.37. In our study, we found lower SPF values, ranging from 2.14 to 3.95, regarding the deseeded pod extracts. Even so, similar SPF values of 18.19 and 13.07 were obtained from CSE2 and CSH2, respectively. A high SPF value (32.44) was also observed with the leaf extract, CSE4 (Table S3). Furthermore, we determined even higher SPF values with carob seed extract fractions CSE2.1, CSE2.2, and CSE2.4 of 31.97, 69.87, and 25.06, respectively. To the best of our knowledge, this is the first study to examine the photoprotective effect of carob extracts on UVB-treated fibroblasts. We showed an increase in the viability of fibroblasts exposed to UVB irradiation that had been pretreated with CSE2 (Figure 4A), which might be related to its high SPF value. In addition, its fraction CSE2.2 showed even greater photoprotective qualities in both assays (Figure 7).

In the present study, a significant inhibition of tyrosinase activity was observed, mainly by the aqueous methanolic seed extracts from both cultivars, and to a lesser extent by the deseeded pod extract from Heraklion (Figure 5A). The hexane seed extracts were inactive in this assay. Following fractionation, the best antityrosinase activity was observed with the fractions partitioned with ethyl acetate and butanol (Figure 8A,B). A similar though less intense inhibitory activity against tyrosinase has been reported for a Turkish carob ethanolic seed extract [79]. In a previous study of the antimelanogenic properties of *C. siliqua* ethanolic leaf, bark, and fruit extracts, monophenolase activity was found to be potently inhibited by all extracts, while diphenolase activity was inhibited mainly by the leaf extract [80], in agreement with our observations regarding the aqueous methanolic leaf extract from the Imera cultivar (Elounda; Table S3). Comparable inhibitory effects have been also reported with carob leaf methanolic and aqueous extracts from Turkish plants [56].

Though the inhibitory effects against collagenase activity were not so intense, statistically significant reduction in the activity of this enzyme was mainly observed with the leaf extract (CSE4; Table S3) and to a lesser extent with seed extract CSE2 and deseeded pod extract CSH1 (Figure 5B). However, upon fractionation, *C. siliqua* pod and seed extract fractions CSE1.2, CSE2.2, and CSE2.3 were found to be very active, with CSE1.2 and CSE2.2 even attenuating collagenase activity to a similar extent to the reference compound (EGCG) (Figure 8C,D). Even though locust bean gum, a polysaccharide from *C. siliqua* seed, has been found to inhibit collagenase [81], as far as we know, this is the first study assessing the effect of carob extracts on collagenase activity.

Natural products’ interference with AGE formation is usually studied as a means to counteract diabetic complications; however, it is also important for cosmetics [82]. Our findings regarding the ability of all aqueous-methanol carob extracts, from both Cretan cultivars, as well as their fractions to inhibit glucose-induced AGE formation were very promising, since CSE1 and CSE2 had activities comparable to the positive control (quercetin; Figure 5C), whilst fraction CSE1.3 completely eliminated AGE formation (Figure 8E). Again, hexane extracts were found to be less active (Figure 5C), while the leaf extract CSE4 (Table S3) was highly active, in agreement with another study in the literature [83].

**Scheme 1:**
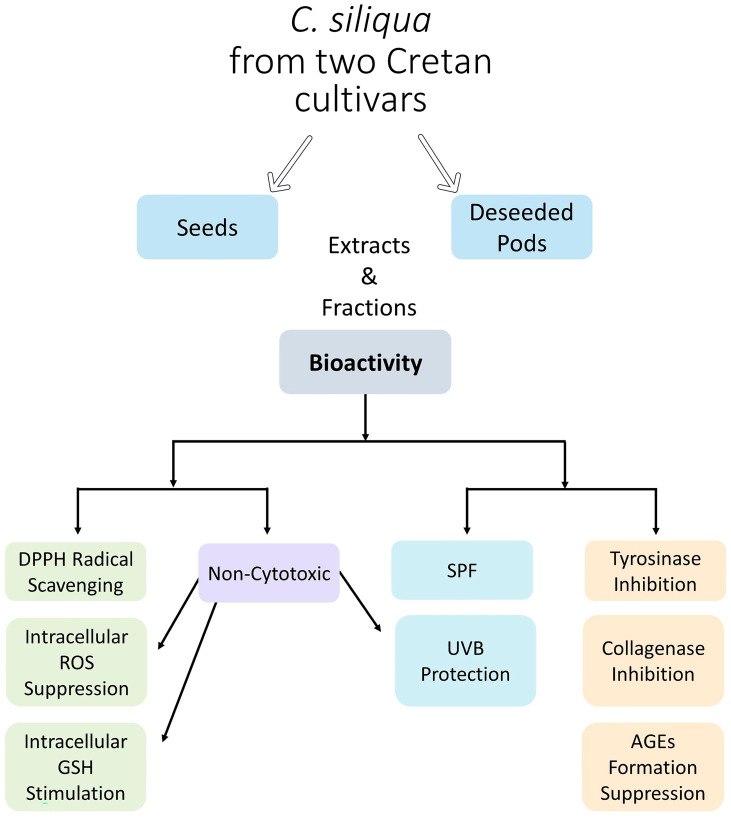
Graphical representation summarizing the findings of the present study.

All the above activities characterizing carob extracts and studied either on human skin fibroblasts in vitro or in cell-free assay systems were, for the first time, investigated from a perspective aiming at the putative use of these extracts in cosmetics. Indeed, the cumulative capacity of these extracts for scavenging free radicals, suppressing ROS, stimulating GSH, inhibiting tyrosinase and collagenase activities, blocking glucose-induced AGEs, and in certain cases counteracting UVB effects (Scheme 1) makes them ideal candidates for skin care products.

## 4. Materials and Methods

### 4.1. Plant Material

The plant material was collected from two different places on the island of Crete. *C. siliqua* E (CSE) was collected from trees belonging to the Imera cultivar in the village Pines in the area of Elounda, Prefecture of Lasithi (coordinates L:35.278545, A:25.715529) with the permission of the local producer Mr. George Drakonakis. This plant was botanically identified by Prof. K. Kalantidis, Department of Biology, University of Crete, Greece, to be used for DNA isolation and sequencing, and a voucher specimen was deposited in the Department Herbarium. *C. siliqua* H (CSH) was collected from trees belonging to the Tylliria cultivar, in the village Melidochori, Prefecture of Heraklion, (coordinates L.35119302, A25.101570) with the permission of the local producer Mr. Kostas Karatzis. Both plant samples were collected during September 2022. They were naturally dried (in the shade and in a well-ventilated environment), ground with a laboratory mill (particle size approx. 1 mm), and stored in the darkness at room temperature.

### 4.2. Extraction and Phytochemical Analysis

#### 4.2.1. Preparation of CSE and CSH Extracts and Fractions

The above powdered plant materials (fruit: 5 kg; seeds: 350 g) of each *C. siliqua* cultivar were extracted with 30% aqueous MeOH for three times to yield CSE1 and CSH1 (from deseeded fruits of each cultivar) and CSE2 and CSH2 (from the seeds of each cultivar), according to the method of Tsiftsoglou et al. [84] with a few modifications. Alternatively, seeds were extracted with a n-hexane as solvent to yield CSE3 and CSH3 (see Table 1). CSE1 and CSE2 extracts from the Imera cultivar, having shown interesting biological activities, were subjected to serial partition as follows [84]: the extracted solutions were concentrated, then suspended in 1L of boiling water and partitioned with different solvents of increasing polarity, namely n-hexane, ethyl acetate (EtOAc), and butanol (BuOH). The fractions corresponding to n-hexane were designated as CSE1.1 and CSE2.1, the ones corresponding to EtOAc as CSE1.2 and CSE2.2, and the ones corresponding to BuOH as CSE1.3 and CSE2.3, while the water-soluble residues were designated as CSE1.4 and CSE2.4 (see Table 7).

#### 4.2.2. Essential Oil Extraction

The oil extraction was obtained from 1 kg of fresh whole *C. siliqua* pods (with seeds) via hydrodistillation over 3 h using a Clevenger-type apparatus [85]. The essential oils from CSE and CSH were then diluted with 2 mL of n-hexane and filtered through anhydrous sodium sulfate to remove water traces. The resulting essential oils were stored at 4 °C. The oil content was estimated in mL/100 g (dry weight of the plant material).

GC–MS analysis of the isolated essential oils was performed on a Shimadzu GC-2030 Nexis gas chromatograph (Shimadzu Corporation, Kyoto, Japan) coupled with a Shimadzu GCMS-QP 2020 NX mass-selective Quadrupole Mass Spectrometer as detector with the appropriate data system. The GC was equipped with a Grob-type split-splitless injector; the fused silica capillary column (Mega-5 HT with 0.25 μm film thickness, 30 m × 0.25 mm i.d.) was directly coupled to the ion source. Helium was used as a carrier gas with a back pressure of 0.8 Atm. The injector temperature was 250 °C and the oven temperature program started at 50 °C for 5 min and then increased at a rate of 5 °C/min up to 150 °C, retained at this temperature for 10 min and increased again at a rate of 5 °C/min up to 280 °C, where it remained for 20 min. The scanning range was 30–700 *m*/*z*. A GS-MS detection electron ionization system was used with ionization energy of 70 eV.

The quantification of the components was based on the total number of fragments (total ion count) of the metabolites, as detected by the mass spectrometer. The identification of the chemical components was carried out based on the retention time of each component (Rt) compared with that of commercially available compounds by analyzing their mass spectra through the use of the NIST21, NIST107, and PMW_TOX2 mass spectra libraries [86] as well as through a comparison with the literature data [87]. A calculation of retention indices was performed in accordance with the work of Van den Dool and Kratz [88], in comparison to the retention times of standard hydrocarbons (C8–C40). When necessary, co-injection with standard compounds was also carried out.

#### 4.2.3. Fatty Acid Composition

The lipid fraction was extracted with n-hexane in a Soxhlet apparatus for 8 h [89]. The total fatty acid composition of the lipid fraction was determined [90]. The identification was performed by comparing the retention times with those of a standard mixture of FAME. The FAMEs were then subjected to a gas chromatograph equipped with a flame ionization detector (GC-FID, Thermoscientific-Trace GC Ultra, Mississauga, ON, Canada) with an injector volume of 1 µL. Initially, the oven temperature was kept at 120 °C for 1 min, and then gradually raised at a scale of 4 °C/min to 240 °C. The inlet as well as detector temperatures were set at 230 and 250 °C and the carrier gas used was helium at a flow rate of 1 mL/min. The FAME peaks were deduced by comparing with the retention times of a standard mixture. Percentages of FAMEs were attained by computing the peak areas using the direct normalization process.

#### 4.2.4. Sugar Determination

Samples were analyzed for mono- and disaccharides using high-performance liquid chromatography (HPLC-RI) according to the AOAC method 982.14 [91]. Sugars were extracted into 50% ethanol; the extract was passed through a C18 Sep-Pak cartridge and then filtered through a 0.45 mm nylon disc. The separation was performed with mobile-phase distilled water at a flow rate of 1.0 mL/min, and the sample injection volume was 20 μL. The stock solutions with sugar (glucose, fructose, sucrose, maltose, and lactose) concentrations of 0.1 mg/mL were injected into an HPLC Lab Alliance Series III pump equipped with Clarity software and a Shodex RI Detector was used using a C18 ODS1 Spherisorb with a 10µm column that measured 250 mm × 10 mm, a flow rate of 1.4 mL/min, isocratic mobile-phase acetonitrile/water at a ratio of 75:25, a temperature of detector and column of 30 °C, and a volume of injection of 5 μL.

#### 4.2.5. Spectroscopic NMR Data

The ^1^H-NMR spectra were recorded in CD_3_OD and CDCl_3_ using Bruker 500 MHz spectrometers. The chemical shifts are provided in δ (ppm) values relative to TMS (3.31 ppm CD_3_OD; 7.26 ppm CDCl_3_).

### 4.3. Cell Cultures and Cell Lines

Normal human foreskin fibroblast cell strain AG01523 (Coriell Institute for Medical Research, Camden, NJ, USA) and two human cancer cell lines, epidermoid carcinoma A431 and fibrosarcoma HT-1080 (both from American Type Culture Collection; ATCC, Rockville, MD, USA), were cultured in Dulbecco’s Modified Eagle Medium (DMEM; PAN Biotech GmbH, Aidenbach, Germany). The medium was supplemented with antibiotics, 100 IU/mL of penicillin, and 100 μg/mL of streptomycin (Biosera, Nuaille, France), as well as 15% or 10% (*v*/*v*) fetal bovine serum (FBS; Gibco BRL, Invitrogen, Paisley, UK).

The cell cultures were incubated in an environment of 5% CO_2_ with appropriate humidity at 37 °C and were subcultured routinely using a trypsin (0.25–0.3% *w*/*v*; Gibco BRL)–citrate solution. Cell counting was performed using a Coulter counter (Beckman Coulter Diagnostics, Brea, CA, USA) after trypsinization and suspension in an isotonic diluent.

### 4.4. MTT Assay: Assessment of Cytotoxicity

Cytotoxicity was estimated using a modification of the MTT assay [92]. More specifically, AG01523 fibroblasts, A431 epidermoid carcinoma, and HT-1080 fibrosarcoma cells were plated in 96-well flat-bottomed transparent microplates in DMEM with appropriate serum conditions (15% and 10% FBS, respectively) to ensure cell adhesion. The seeding density was approximately 8000 cells/well for the fibroblasts and 3500 cells/well for the cancer cells. After 18 h, additional medium was added along with test extracts to achieve serial dilutions. Since the test extracts were dissolved in dimethylsulfoxide (DMSO; Sigma, St. Louis, MO, USA), corresponding dilutions of DMSO were used as controls, while doxorubicin hydrochloride (Sigma) served as the positive control. Following an incubation period of 72 h, the medium was replaced by serum-free, phenol-red-free DMEM (PAN Biotech GmbH) containing 1 mg/mL of MTT (Sigma). After an additional four-hour incubation, the insoluble purple formazan crystals were solubilized in 2-propanol and the absorbance was measured using a Spark multimode microplate reader (Tecan Group Ltd., Männedorf, Switzerland) at a wavelength of 550 nm (reference wavelength 690 nm). The absorbance of the MTT-formazan was proportional to the viable cells in each well. The results, regarding the normal fibroblasts, are presented as the highest noncytotoxic concentrations evaluated. On the other hand, for the cancer cell lines, the half-maximal inhibitory concentration (IC_50_) and maximal degree of inhibition were calculated where applicable. Each experiment was performed in quadruplicate, and the results represent the mean of three independent experiments.

### 4.5. Antioxidant Activity

#### 4.5.1. DPPH Assay: Radical Scavenging

The radical-scavenging activity of the samples (extracts/fractions) against the DPPH radical was assessed using the previously described DPPH method [93] with slight modifications. In a clear 96-well microplate, 40 μL of DMSO, 10 μL of sample (test extract) and 50 μL DPPH solution (1mM in absolute ethanol, freshly prepared) were added. The extracts were diluted appropriately in DMSO to yield final concentrations ranging from 2000 to 4 μg/mL. DMSO (vehicle for stock extract preparation) served as the negative control and Trolox at 200 μM, a water-soluble analog of vitamin E, served as the positive radical scavenger by adding either 50 μL of DMSO or Trolox and 50 μL of DPPH solution. The DPPH solution was added briefly before measuring the absorbance at 520 nm using a FLUOstar Optima microplate reader (BMG Labtech, Ortenberg, Germany). Scavenging of the DPPH radical by antioxidant compounds leads to a loss of the deep purple color and, thus, the ability of the radical to absorb at 520 nm. The changes in color (alluding to the DPPH radical absorbance) were monitored at regular intervals for four hours after its addition. Experiments were conducted with a minimum of two individual repeats. In every repeat, each sample was added twice. The results are expressed as a percentage of the negative control corresponding to the absorbance of the DPPH radical. None of the extracts and fractions inherently absorbed at 520 nm; thus, normalization with the absorbance of the blank samples was not necessary. IC_50_ values (μg/mL), corresponding to exact concentrations required to scavenge 50% of the available DPPH radicals, were calculated when possible.

#### 4.5.2. DCFH-DA Assay: Intracellular Reactive Oxygen Species (ROS) Levels

The cells were plated in appropriate serum conditions in clear-bottomed black 96-well microplates. Once confluent, the medium was discarded, and the test extracts were added in serum-free medium at various noncytotoxic concentrations. DMSO-treated cell cultures served as a negative control and Trolox was used as a positive control at a final concentration of 100 μΜ. Following an overnight incubation, DCFH-DA (Sigma) was added at a final concentration of 10 μM in serum-free DMEM. After 30 min, fluorescence emission, corresponding to DCF formation, was monitored at 520 nm following excitation at 485 nm, using a FLUOstar Optima microplate reader, for six hours. The experiments were repeated three times. Each time, the samples were added in quadruplicate. Furthermore, to assess the impact of the test extracts on the stimulated intracellular ROS levels, 160 μM of H_2_O_2_ in serum-free medium was added, after the 30 min incubation with DCFH-DA, and the fluorescence emission was measured in the same manner. Two independent repeats of the H_2_O_2_-provoked ROS production were conducted. The results, in both cases, are expressed as a percentage of the DMSO-treated cells.

#### 4.5.3. Cellular Glutathione Levels

An adaptation of the microplate assay based on the fluorescent probe MCB was used for the assessment of the glutathione (GSH) levels in human skin fibroblasts [94,95]. Briefly, AG01523 cells were plated at a 1:2 split ratio in clear-bottomed black 96-well microplates and allowed to attach overnight. Then, the test extracts and fractions as well as the corresponding controls were administered solubilized in phenol-red-free, serum-free DMEM, and the cells were incubated for 20 h either under normal oxygen tension (see above cell culture and cell lines) or in an environment of 2% O_2_ and 5% CO_2_ at 37 °C in an InVivo2 400 hypoxia workstation (Ruskinn Technology Ltd., Bridgend, UK), since hypoxia has been shown to minimize GSH levels in various cell types [69,70]. At the end of the incubation period, the medium was replaced with 5 μM of mCB (MedChemExpress, Monmouth Junction, NJ, USA) diluted in Hanks’ balanced salt solution (HBSS) for a further 4 h, and fluorescence was recorded in a Spark (Tecan) plate reader using an excitation wavelength of 380 nm and emission wavelength of 480 nm. N-acetycysteine (NAC) at 10 mM was used as a positive control [96], vehicle (DMSO) at a concentration corresponding to the extracts’ concentration as a negative control, while treatment of the cells with 8 mM N-ethylmaleimide (NEM) known to deplete cellular GSH helped to discern nonspecific background labelling [97].

### 4.6. Western Blot Analysis

The test extracts were added to confluent human fibroblasts in medium supplemented with 0.1% FBS for selected stimulation time spans right before the protein extraction, with Laemmli sample buffer containing protease and phosphatase inhibitors (Sigma) and a scrapper. The cell lysates were briefly heated at 100 °C, sonicated and centrifuged to collect the clarified supernatant, and stored at −80 °C. The proteins were separated using SDS-PAGE (10% Bis-Tris polyacrylamide gels) and transferred to polyvinylidene fluoride (PVDF) membranes (Thermo Scientific, Rockford, IL, USA). The membranes were incubated overnight with the appropriate primary antibodies upon blocking with 5% *w*/*v* nonfat milk in TBS-T (TBS supplemented with 0.05% Tween-20 buffer) for an hour. The primary antibodies utilized, purchased from Cell Signaling Technology (Beverly, MA, USA), were specific to phospho-Akt (Ser473) and phospho-AMPKα (Thr172), respectively. Subsequently, the membranes were washed three times with 5% *w*/*v* nonfat milk in TBS-T, before probing with the appropriate species-specific horseradish-peroxidase-conjugated secondary antibody (Sigma). Finally, after washing twice with 5% *w*/*v* nonfat milk in TBS-T and once with TBS-T alone, the immunoreactive bands were visualized with chemiluminescence using a horseradish peroxidase substrate (Immobilon Crescendo Western HRP substrate, Merck Millipore) and captured using a LAS-4000 luminescent image analyzer (Fujifilm Manufacturing USA Inc., Greenwood, SC, USA). To validate equal loading probing with a mouse monoclonal antipan-actin antibody (NeoMarkers, Lab Vision, Fremont, CA, USA) was performed, either following the stripping of the membrane, or in a parallel run under identical conditions.

### 4.7. Photoprotective Activity

#### 4.7.1. Protective Activity against UVB Irradiation

Human fibroblasts were plated at a 1:2 ratio (approximately 8000 cells/well) in normal culture conditions to ensure proper cell adherence. Once confluent, the medium was changed to phenol-red-free DMEM supplemented with 15% serum and the test extracts/fractions. Following a five-hour incubation, the medium was discarded and replaced by PBS containing the proper concentrations of the samples and the cells were irradiated in a UV box for 11 min (corresponding to an irradiation dose of 475 mJ/cm^2^). The employed UVB lamp had an emission spectrum of 280–360 nm with a peak at 306 nm. Subsequently, the PBS was changed once more to the serum-enriched phenol-red-free DMEM containing the samples used before, and the cells were incubated in proper culture conditions for 72 h. DMSO-treated cells, diluted correspondingly, were used as non-sample-treated controls since the extracts/fractions were dissolved in DMSO. The cells treated in an identical manner but not irradiated served as non-UVB-treated controls. Finally, their viability was assessed using the Neutral red method, as described [28]. In short, the medium was emptied, and neutral red was added in serum-free, phenol-red-free DMEM (final concentration 0.0075% (*w*/*v*)) for 4 h. The uptake of the neutral red dye by viable cells was determined after washing with PBS and dissolving with a solution of ethanol and water acidified with acetic acid. The neutral red fluorescence emission was measured at 645 nm following excitation at 530 nm using a Spark multimode microplate reader (Tecan). The experiments were conducted in triplicate, and the results are presented as a percentage of the DMSO-treated, non-UVB-treated negative control of three separate repeats.

#### 4.7.2. UVB Absorbance Scan and SPF Assessment

The in vitro sun protection factor (SPF) of the extracts was determined using an adaptation of a previously described method [29]. The samples (extracts and fractions) were properly diluted in PBS (200 μg/mL) and their absorption spectra (250–450 nm) were measured using a U-2900 UV–Vis spectrophotometer (Hitachi High-Technologies Corporation, Tokyo, Japan) every 5 nm against a blank of PBS. Quartz cuvettes were utilized. Subsequently, the SPF was calculated using the following equation:

(1) $$SPF=CF\times \sum_{290}^{320}EE(\lambda )\times I(\lambda )\times Abs(\lambda )$$

where CF = correction factor (=10), EE(λ) = erythemal effect spectrum, I(λ) = solar intensity spectrum, and Abs(λ) = absorbance of the sample. Sayre [98] determined the values of EE(λ) × I(λ), which are constants.

### 4.8. Inhibitory towards on Tyrosinase, Collagenase, and AGE Formation

#### 4.8.1. Tyrosinase Inhibition

Tyrosinase catalyzes the oxidation of L-DOPA to dopaquinone and eventually to dopachrome. Mushroom tyrosinase inhibitory activity was assessed using L-DOPA as substrate and by measuring the absorbance of the forming dopachrome, as previously reported [92], with slight modifications. Samples properly diluted in phosphate buffer (0.1M KH_2_PO_4_/K_2_HPO_4_, pH 6.5) were incubated along with a mushroom tyrosinase (Sigma) solution (100 U/mL in reaction buffer) in a 96-well microplate at room temperature for 10 min. The final concentration of the samples was 200 μg/mL. DMSO, in the corresponding dilution, served as the negative control, given that it was used to dissolve the test samples, whilst kojic acid (Apollo Scientific, Stockport, UK) at 0.1 mM served as a positive control. In a similar manner, a blank for each sample/control was prepared in the absence of tyrosinase. Afterwards, the substrate (L-DOPA; Merck KGaA, Darmstadt, Germany) was combined and the reaction was immediately monitored by measuring the absorbance at 475 nm (37 °C) with a Spark multimode microplate reader (Tecan). The percentage of the enzyme inhibition was calculated according to the following equation:

(2) $$\%Inhibition=\frac{\left[\left(C-Cb\right)-\left(S-Sb\right)\right]}{\left(C-Cb\right)}\times 100$$

where C: control, Cb: blank control, S: sample, and Sb: blank sample. Each sample was tested in duplicate, and the results are presented as the mean ± SD of two independent experiments.

#### 4.8.2. Collagenase Activity

To determine the effect of the samples on collagenase activity, *Clostridium histolyticum*-derived collagenase (Sigma) and the fluorogenic substrate Dabcyl-Gaba-Pro-Gln-Gly-Leu-Glu (EDANS)-Ala-Lys-NH_2_ (TNO211; Calbiochem) were utilized. TNO211 is a nonfluorescent peptide in its entirety, due to fluorescence resonance energy transfer (FRET), that can be detected using fluorometry when cleaved by collagenase since the fluorophore, EDANS, is released from its quencher, Dabcyl [99]. Therefore, in a 96-well plate, the samples were incubated for 15 min, at 37 °C, in the presence of collagenase. Collagenase was diluted in Tris-HCl buffer supplemented with Ca^2+^ (pH: 7,5) to achieve a final concentration of 50 μg/mL. Upon incubation, the substrate properly diluted in reaction buffer was added to obtain a final concentration of 10 mM. The fluorescence emission was then monitored at 480 nm, following excitation at 340 nm, using a FLUOstar Optima microplate reader. DMSO, in the corresponding concentrations of the samples, served as the negative control and EGCG at 1 mM served as the positive control [100]. The fluorescence of a blank sample, containing the buffer and the substrate but no enzyme, was also measured. The percentage of collagenase activity was calculated using the following equation:

(3) $$\%Collagenaseactivity=\frac{\left(S-B\right)}{\left(C-B\right)}\times 100$$

where S: sample, B: blank, and C: control. The experiment was conducted twice; each time, the samples were added in duplicates. The results are demonstrated as mean ± SD.

#### 4.8.3. In Vitro Glycation Assay

An adaptation of a previously described method [101] was used to assess the putative inhibitory activity of the carob extracts against the formation of advanced glycation end products (AGEs). Briefly, bovine serum albumin (BSA) fraction V (Sigma) diluted in 50 mM phosphate buffer pH 7.4 was added to the carob extracts in the presence or absence of D-glucose (Sigma). The final concentrations of the reagents in the solution were 400 μg/mL BSA, 200 μg/mL extract, and 200 mM glucose. The mixture was heated to 55 °C for 60 h, and then 100 μL aliquots of each sample were subjected to protein precipitation through the addition of 10 μL of 100% *w*/*v* trichloroacetic acid and centrifugation (15,000 rpm; 10 min; 4 °C). The supernatant was discarded and the pellet was resuspended in 400 μL of alkaline (pH 10) PBS. The fluorescence of the final solution was monitored; the reaction was recorded in a Spark (Tecan) plate reader using an excitation wavelength of 370 nm and emission wavelength of 430 nm. Quercetin at a concentration of 200 μg/mL (approx. 662 μM) was used as a positive control [102].

### 4.9. Statistical Analysis

The results are presented as the mean ± standard deviation (SD) of at least two independent experiments, unless indicated otherwise. Statistical differences were evaluated by performing Student’s *t-*test analysis and values of *p* < 0.05 were considered statistically significant. Statistical analysis and graph creation was performed either with GraphPad Prism 5 software (GraphPad Software, San Diego, CA, USA) or with Microsoft^®^ Excel^®^ 2019 MSO (Version 2306 Build 16.0.16529.20164) 64-bit.

## 5. Conclusions

Deseeded pods and seeds from two of the most common *C. siliqua* cultivars encountered on the island of Crete were subjected to phytochemical analysis and to an evaluation of their biological activities. While their phytochemical profile was found to fit within the wide array of compositions reported in the literature from plants of this genus all over the world, certain features were identified that characterized each cultivar and matched their current uses. Moreover, the biological properties of their extracts, including antioxidant capacity in a cell-free assay and various cell-based ones; tyrosinase- and collagenase-inhibitory activities; ability to block glucose-induced AGEs; and—in certain cases—UVB absorbance and photoprotective activities; along with the lack of any cytotoxicity make them suitable candidates for use in cosmetics. For example, based on tyrosinase inhibition, carob extracts may be used for skin-whitening applications, while their UV shield and photoprotective capacity may lead to their use in sunscreen products. Bearing in mind that seeds are considered by-products of carob powder production, the superior biological activities of seed extracts observed in this study make them even more promising for valorization and upcycled cosmetics.

## Figures and Tables

**Table 1:** Carob extracts.

Cultivar (Area)	Sample Name	Plant Part	Solvent
Imera (Elounda)	CSE1	Fruit	30% aqueous methanol
	CSE2	Seeds	30% aqueous methanol
	CSE3	Seeds	n-hexane
Tylliria (Heraklion)	CSH1	Fruit	30% aqueous methanol
	CSH2	Seeds	30% aqueous methanol
	CSH3	Seeds	n-hexane

**Table 2:** Qualitative and quantitative composition of the essential oils from *C. siliqua*.

Νο	Ret. Time	Compounds	RI ^1^	RIlit ^2^	CSE	CSH
1	7.817	Hexanoic acid	972	973	11.16 ± 0.01	14.10 ± 0.02
2	11.014	2-Nonanone	1091	1090	n.d. ^3^	4.60 ± 0.04
3	11.307	2-Nonanol	1098	1098	n.d.	1.58 ± 0.00
4	11.452	Nonanal	1100	1100	n.d.	1.04 ± 0.01
5	12.038	2Ζ-Nonen-1-al	1149	1149	n.d.	1.39 ± 0.02
6	12.443	2E, 6Z Nonadienol	1162	1163	n.d.	0.60 ± 0.00
7	13.347	Octanoic acid	1170	1171	13.10 ± 0.24	26.73 ± 0.93
8	13.955	2-Decanone	1192	1192	n.d.	0.66 ± 0.00
9	16.048	Nonanoic acid	1270	1270	5.88 ± 0.04	3.63 ± 0.02
10	16.357	2E, 4Z Decadienal	1293	1293	n.d.	2.64 ± 0.05
11	16.810	2-Undecanone	1296	1294	n.d.	5.08 ± 0.15
12	17.025	Carvacrol	1298	1299	1.53 ± 0.05	1.61 ± 0.20
13	17.787	2E, 4E Decadienal	1314	1316	n.d.	3.33 ± 0.50
14	18.393	9-Decenoic acid	1360	1361	0.61 ± 0.00	n.d.
15	18.709	Decanoic acid	1364	1366	3.32 ± 0.70	2.99 ± 0.05
16	22.155	2-Tridecanone	1496	1496	0.72 ± 0.00	3.76 ± 0.50
17	23.740	Dodecanoic acid	1566	1566	4.02 ± 0.25	n.d.
18	25.820	epi-α-Cadinol	1642	1640	n.d.	2.52 ± 0.15
19	26.351	6Z-Pentadecen-2-one	1668	1668	3.25 ± 0.15	4.25 ± 0.10
20	26.679	Tetradecanol	1674	1672	1.55 ± 0.29	n.d.
21	26.962	2-Pentadecanone	1696	1697	9.75 ± 0.33	7.16 ± 0.05
22	27.137	Heptadecane	1700	1700	4.34 ± 0.05	1.71 ± 0.00
23	28.330	Tetradecanoic acid	1764	1763	4.10 ± 0.08	n.d.
24	30.038	Hexahydrofarnesyl acetone	1836	1838	4.87 ± 0.02	n.d.
25	30.630	Z-Z-9,12-Octadecanoic acid	2132	2133	9.58 ± 0.75	5.48 ± 0.05
26	30.955	Oleic acid	2142	2142	12.66 ± 0.80	3.65 ± 0.05
27	31.307	1-Docosene	2189	2189	7.82 ± 0.54	n.d.
		Total (%)			98.26	98.51
		Percentage of yield			0.10%	0.11%

^1^ Retention indices as determined on a Mega-5 HT MS capillary column using a homologous series of n-alkanes (C9-C25); ^2^ RIlit: Retention indices according to literature; ^3^ n.d.: not detected.

**Table 3:** Fatty acid (% of total) from *C. siliqua*.

Fatty Acids, %		CSE	CSH
Lauric	C12:0	0.08 ± 0.00	0.05 ± 0.00
Myristic	C14:0	0.24 ± 0.00	0.42 ± 0.00
Palmitic	C16:0	15.65 ± 0.10	17.90 ± 0.10
Palmitoleic	C16:1, cis-9	0.15 ± 0.00	0.05 ± 0.00
Palmitoleic	C16:1, trans-9	0.99 ± 0.00	0.08 ± 0.00
Heptadecanoic	C17:0	0.06 ± 0.00	0.02 ± 0.00
Heptadecenoic	C17:1, cis-10	0.35 ± 0.01	0.21 ± 0.01
Stearic	C18:0	4.17 ± 0.05	3.00 ± 0.01
Oleic	C18:1, ω-9	38.38 ± 0.30	32.10 ± 0.20
Vaccenic	C18:1, cis ω-7	2.00 ± 0.00	1.25 ± 0.00
Linoleic	C18:2, ω-6	35.61 ± 0.42	43.05 ± 0.50
Arachidic	C20:0	0.94 ± 0.00	0.85 ± 0.00
Linolenic	C18:3, ω-3	0.64 ± 0.00	0.42 ± 0.00
Eicosenoic	C20:1, cis ω-9	0.28 ± 0.00	0.20 ± 0.00
Behenic	C22:0	0.36 ± 0.00	0.30 ± 0.00
Lignoceric	C24:0	0.10 ± 0.00	0.10 ± 0.00
Σ-SFA		21.60	22.74
Σ-MUFA		42.15	33.89
Σ-PUFA		36.25	43.47
Total ω3		0.64	0.42
Total ω6		35.61	43.05

**Table 4:** Sugar content of two Cretan carob pods.

	CSE	CSH
Fructose	7.90 ± 0.02	7.0 ± 0.01
Glucose	4.40 ± 0.01	3.20 ± 0.01
Sucrose	33.20 ± 0.50	39.50 ± 0.30
Maltose	n.d.^1^	n.d.
Lactose	n.d.	n.d.
Total sugars (g/100g)	45.5	49.7

^1^ n.d.: not detected.

**Table 5:** Sun protection factor (SPF) of *C. siliqua* extracts.

Cultivar (Area)	Sample	SPF
Imera (Elounda)	CSE1	3.95
	CSE2	18.19
	CSE3	8.13
Tylliria (Heraklion)	CSH1	2.14
	CSH2	13.07
	CSH3	1.07

**Table 6:** Carob fractions.

Cultivar (Area)	Extract	Sample Name	Partition Solvent
Imera (Elounda)	CSE1	CSE1.1	n-hexane
		CSE1.2	ethyl acetate (EtOAc)
		CSE1.3	n-butanol (BuOH)
		CSE1.4	aqueous residue
	CSE2	CSE2.1	n-hexane
		CSE2.2	ethyl acetate (EtOAc)
		CSE2.3	n-butanol (BuOH)
		CSE2.4	aqueous residue

**Table 7:** Sun protection factor of CSE2 fractions.

Cultivar (Area)	Sample	SPF
Imera (Elounda)	CSE2.1	31.97
	CSE2.2	69.87
	CSE2.3	25.06
	CSE2.4	6.37

## Data Availability

Data presented in this study are contained within the article or the supplementary material.

## Supplementary Materials

The supporting information can be downloaded at: https://www.mdpi.com/article/10.3390/ijms241512104/s1.
