# Supplementary material for: Biological Activities of Ceratonia siliqua Pod and Seed Extracts: A Comparative Analysis of Two Cretan Cultivars
Source: Int J Mol Sci. 2023 Jul 28;24(15):12104. doi: 10.3390/ijms241512104 (PMC10418674; doi:10.3390/ijms241512104)
Supplement: Supplementary file 1 [file ijms-24-12104-s001.zip › ijms-2509485-supplementary.pdf]

International Journal of
Molecular Sciences

MDPI

Article

Biological Activities of *Ceratonia siliqua* Pod and Seed Extracts: A Comparative Analysis of Two Cretan Cultivars

Dafni-Alexandra Kavvoura [1], Michalis K. Stefanakis [2], Dimitris Kletsas [1], Haralambos E. Katerinopoulos [2] and Harris Pratsinis [1,*]

[1] Laboratory of Cell Proliferation and Ageing, Institute of Biosciences and Applications, NCSR "Demokritos", 15341 Athens, Greece; dafnikavvoura@gmail.com (D.-A.K.); dkletsas@bio.demokritos.gr (D.K.)
[2] Laboratory of Organic Chemistry, Department of Chemistry, University of Crete, 70013 Heraklion, Greece; michstefanakis@yahoo.gr (M.K.S.); katerinc@uoc.gr (H.E.K.)
* Correspondence: hprats@bio.demokritos.gr; Tel.: +30-2106503572 or +30-2106503561

Supplementary information

Table S1. Cell viability following a 24-hour incubation with carob samples.

Cultivar (Area)	Sample	Cell Lines (MTT Assay)				
		Normal Cells	Cancer Cells			
		AG01523	A431		HT-1080	
		Highest Non-Cytotoxic Dose (μg/mL)	IC_{50} ± SD (μg/mL)	Maximal Degree of Inhibition (%)	IC_{50} ± SD (μg/mL)	Maximal Degree of Inhibition (%)
Imera (Elounda)	CSE1	200	>200	N/A	>200	N/A
	CSE2	200	>200	N/A	>200	N/A
	CSE3	200	>200	37 ± 31	>200	N/A
Tylliria (Heraklion)	CSH1	200	>200	N/A	>200	N/A
	CSH2	200	>200	N/A	>200	N/A
	CSH3	200	>200	N/A	>200	N/A

Highest non-cytotoxic doses of carob samples tested on normal fibroblasts. Half maximal inhibitory concentration (IC_{50}) values ± standard deviation (SD) (μg/mL) and maximal degree of inhibition percentages against cancer cell lines[a].
N/A; non-active, indicating that the sample exhibited no inhibitory effect at the highest concentration tested (200 μg/mL).
The results are expressed as mean ± SD of three independent experiments, conducted in quadruplicates. Dimethylsulfoxide (DMSO)-treated cells served as controls.

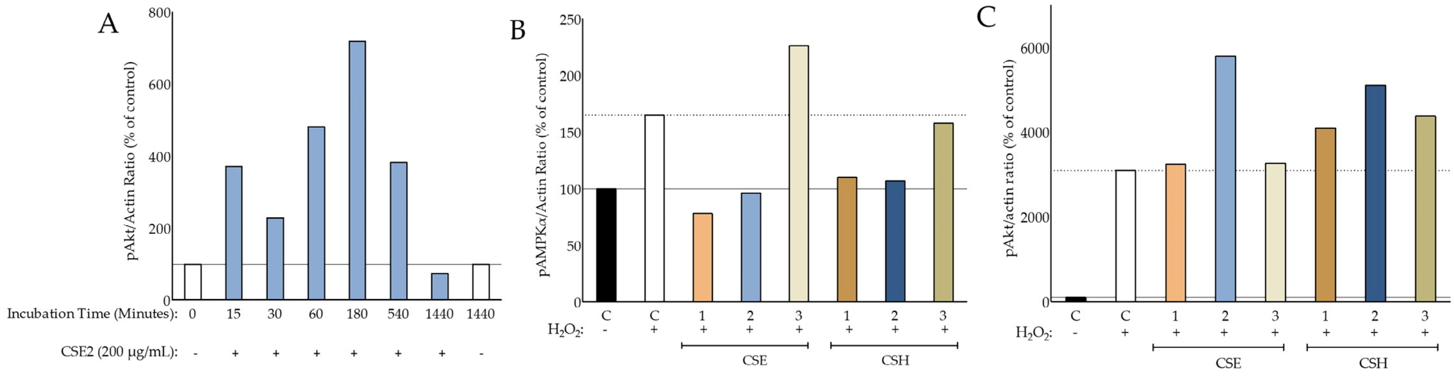

Figure S1. Western blot analysis of carob extracts; illustration of densitometry results. (**A**) Akt phosphorylation upon incubation with CSE2. (**B**) Effects of pre-treatment of 180 minutes with carob extracts on AMPKα phosphorylation of fibroblast treated with H_2O_2 (800 μM) for 45 minutes. (**C**) Effects of pre-treatment of 180 minutes with carob extracts on Akt phosphorylation of fibroblast treated with H_2O_2 (800 μM) for 45 minutes. Densitometry was performed using ImageJ 1.54b.

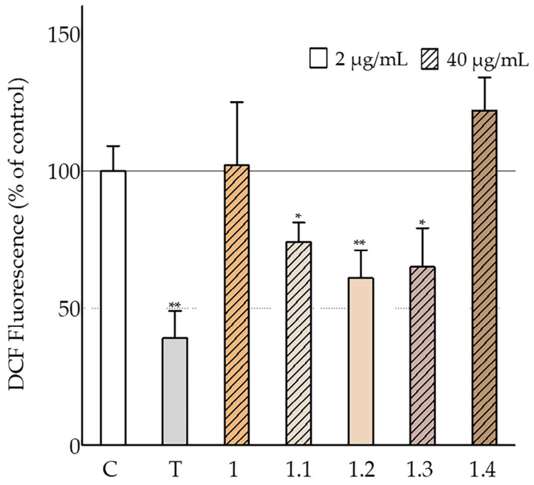

Figure S2. Intracellular (AG01523) antioxidant activity of CSE1 fractions, at lower concentrations, on basal ROS levels, 210 minutes after DCFH-DA addition (*n*=3). DMSO-treated cells served as controls (C) and Trolox (T) as the reference compound. Bars represent mean values ± SD. * $p<0.05$; ** $p<0.01$ (Student's t-test). Control values were set to 100%.

Table S2. Effects of carob fractions on normal and cancer cell viability.

Cultivar (Area)	Sample	Cell Lines (MTT Assay)				
		Normal Cells	Cancer Cells			
		AG01523	A431		HT-1080	
		Highest Non-Cytotoxic Dose (μg/mL)	IC_{50} ± SD (μg/mL)	Maximal Degree of Inhibition (%)	IC_{50} ± SD (μg/mL)	Maximal Degree of Inhibition (%)
Imera (Elounda)	CSE1.1	200	96 ± 1	95 ± 0	87 ± 18	98 ± 0
	CSE1.2	10	28 ± 6	90 ± 5	31 ± 9	97 ± 2
	CSE1.3	200	>200	N/A	135 ± 36	67 ± 15
	CSE1.4	200	>200	N/A	>200	N/A
	CSE2.1	40	62 ± 28	94 ± 4	90 ± 6	99 ± 2
	CSE2.2	200[2], 40[3]	>200	41 ± 12	114 ± 28	76 ± 13
	CSE2.3	200	>200	N/A	>200	21 ± 5
	CSE2.4	200	>200	N/A	>200	N/A

Highest non-cytotoxic doses of carob samples tested on normal fibroblasts. Half maximal inhibitory concentration (IC_{50}) values ± standard deviation (SD) (μg/mL) and maximal degree of inhibition percentages against cancer cell lines[1].

N/A; non-active, indicating that the sample exhibited no inhibitory effect at the highest concentration tested (200 μg/mL).

[1] The results are expressed as mean values ± SD of three independent experiments, conducted in quadruplicates.

[2] Highest cytotoxic concentration in normal serum conditions.

[3] Maximal non-cytotoxic dose in serum-free and low serum conditions.

Table S3. *C. siliqua*, from Imera cultivar (Elounda), 30% aqueous methanolic leaf extract activities (CSE4).

**Cell Viability**	**Cell Line**	**Highest Non-Cytotoxic Dose (μg/mL)**	**IC_{50} ± SD (μg/mL)**	**Maximal Degree of Inhibition (%)**
MTT Absorbance	AG01523	200		
	A431		>200	41 ± 4
	HT-1080		108 ± 11	75 ± 8
**Antioxidant Activity**	**Cell Line**	**Concentration (μg/mL)**	**Maximal Activity (% of Control)**	
DPPH Absorbance		100	37 ± 7	
Intracellular ROS Levels/DCFH-DA Fluorescence		N/D	N/D	
Intracellular Glutathione Levels/MCB Fluorescence	AG01523	200	193 ± 38 (Hypoxia), 126 ± 3 (Normoxia)	
**Photoprotective Activity**	**Cell Line**			
UVB Protection/Neutral Red Fluorescence	AG01523	N/A		
SPF		32.44		
**Inhibition of Tyrosinase, Collagenase and AGEs Formation (200 μg/mL)**				
Tyrosinase Inhibition (%)	53 ± 0			
Collagenase Activity (%)	61 ± 3			
AGEs Formation Inhibition (%)	89 ± 2			

N/A; non-active; N/D; not determined.
